# Figuring it out by yourself: Perceptions of home-based care of stroke survivors, family caregivers and community health workers in a low-resourced setting, South Africa

**DOI:** 10.4102/phcfm.v12i1.2629

**Published:** 2020-10-08

**Authors:** Elsje Scheffler, Robert Mash

**Affiliations:** 1 Division of Family Medicine and Primary Care, Faculty of Medicine and Health Sciences, Stellenbosch University, Cape Town, South Africa

**Keywords:** stroke rehabilitation, community health workers, family caregivers, home-based, low- and middle-income country/ies, South Africa, primary health care, training needs

## Abstract

**Background:**

In less resourced settings, formal rehabilitation services for stroke survivors were often absent. Stroke survivors were referred to community health workers (CHWs) who were untrained in rehabilitation.

**Aim:**

To describe the experience and perceived needs of stroke survivors, their caregivers and CHWs in a context with limited access to and support from formal rehabilitation services.

**Setting:**

The Breede Valley subdistrict, Western Cape, South Africa, a rural, less resourced setting.

**Methods:**

A descriptive exploratory qualitative study. Four focus group interviews were held with purposively selected stroke survivors and caregivers and four with CHWs. A thematic approach and the framework method were used to analyse the transcripts.

**Findings:**

A total of 41 CHWs, 21 caregivers and 26 stroke survivors participated. Four main themes and 11 sub-themes were identified. Because of the lack of knowledge, training and rehabilitation services, the main theme for all groups was having to ‘figure things out’ independently, with incontinence management being particularly challenging. Secondly was the need for emotional support for stroke survivors and caregivers. Thirdly, contextual factors such as architectural barriers and lack of assistive products negatively impacted care and function. Lastly, the organisation of health and rehabilitation services negatively impacted home-based services and professional support.

**Conclusions:**

With appropriate training, the CHWs can be pivotal in the training and support of family caregivers and stroke survivors. Care pathways and the role and scope of both CHWs and therapists in home-based stroke rehabilitation should be defined and restructured, including the links with formal services.

## Introduction

The global stroke burden places an increasing demand on the health and rehabilitation resources in all countries.^1,2,3,4^ Informal family caregivers play an increasingly important role in the continuum of stroke care.^5,6,7,8,9^ Their roles, experiences and needs have been widely recognised, explored and documented.^5,10,11,12,13,14,15,16,17,18,19,20,21^ Caregiver interventions, as part of acute or rehabilitation services, have demonstrated significant improvements in caregiver knowledge and skills and stroke survivor function and a decrease in complications in all country settings.^10,22,23^

In low- and middle-income countries, rehabilitation services are often unavailable or inaccessible, and stroke survivors are discharged home directly from acute care without any caregiver training,^6,10^ highlighting the need for care models that explicitly incorporate caregivers. There is a paucity of information on the experiences and needs of caregivers in low- and middle-income countries, particularly in settings where stroke survivors do not receive formal rehabilitation services. As caregiver training should be based on the needs of caregivers, their needs in these settings should be identified. Existing caregiver training programmes in all settings^24,25,26,27,28,29,30,31,32,33^ have usually been designed in conjunction with formal rehabilitation services and therefore cannot be applied to a community-based setting without such formal rehabilitation services.

In the Western Cape Province, South Africa (SA), stroke mortality and morbidity are higher than the national average.^34,35^ There are no stroke units in the rural districts, and stroke survivors are discharged home from acute care hospitals to untrained family caregivers. Although South African health policy promotes a primary health care approach with a continuum of promotive, preventive, curative, rehabilitative and palliative services; rehabilitation services are fragmented, infrequent and have poor capacity to cope with the service demand.^36^ Despite free healthcare, contextual factors, such as poverty, lack of transport or inaccessible public transport, further limit access to these facilities.^37,38,39,40,41^

Home- and community-based care (HCBC) services are delivered by teams of community health workers (CHWs)^42,43^ led by nurses (home-based care coordinators) who are responsible for conducting assessments and determining treatment plans. The CHWs are lay workers with mostly informal training, specific to the context they work in, and who focus on health promotion, prevention, curative and palliative services. Although they have limited rehabilitation training, CHWs are often the only and or closest healthcare service provider available. Their experiences on providing stroke rehabilitation have not been studied before. The fact that Bryer et al.^8^ advocated for a South African HCBC model targeting caregivers and stroke survivors as well as the local district manager requesting a home-based stroke training programme for CHWs provided the researcher with the opportunity to conduct this study.

The aim of this article is to describe the experiences and perceived needs of stroke survivors, their family caregivers and CHWs in a context with limited access to and support from formal rehabilitation services.

## Methods

### Study design

This study was part of a larger mixed-methods study with the overall aim of developing a home-based stroke rehabilitation programme in the Cape Winelands district of the Western Cape, SA. The situational analysis informing the design and development of the training programme included a concurrent quantitative study reporting on the outcomes of the current HCBC in the district^44^ as well as this qualitative study.

A descriptive exploratory qualitative study using focus group interviews (FGIs) and thematic analysis^45^ was used to describe and explore the experiences and perceived home-based rehabilitation needs of stroke survivors, caregivers and CHWs.

The researcher is a physiotherapist with more than 20 years’ experience in stroke rehabilitation, working in less resourced settings and developing and delivering rehabilitation-related training in these settings to rehabilitation professionals and mid-level and grass-roots level workers. Whilst the researcher had a close working relationship with the therapists in the district, contact with HCBC was limited.

### Setting

**FIGURE 1:**
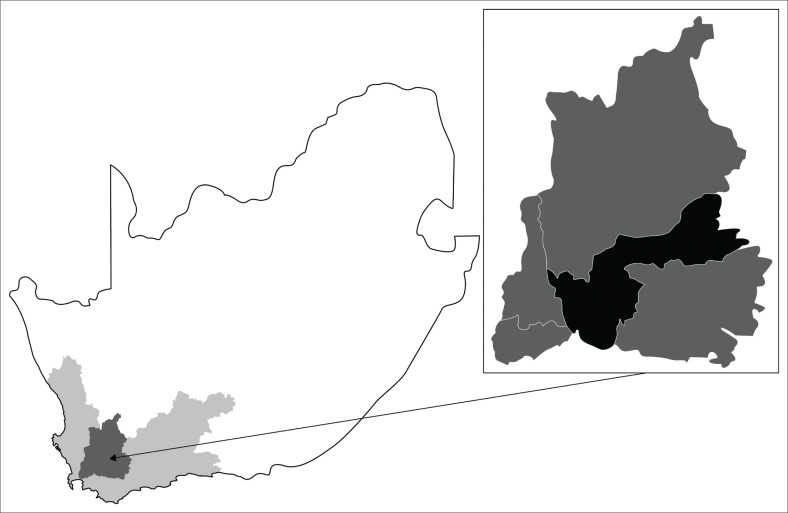
Map of South Africa illustrating the Western Cape province (light grey area), with the Cape Winelands (darker grey area). The insert shows five subdistricts with the study setting, Breede Valley, blacked out.

The Cape Winelands district is a rural district of the Western Cape province (Figure 1). At the time of the study, the rural Cape Winelands district (population of 866 000) recorded more than 600 stroke-related admissions annually in its six public-sector acute hospitals. After an average length of stay of 5 days,^44^ most stroke survivors were discharged home to untrained family caregivers. There was a lack of clinical practice guidelines and pathways.^44^ Rehabilitation services were limited to one multidisciplinary therapy team roving between clinics in a subdistrict. Typically, this team consists of one physio-, occupational and speech therapist, delivering services at selected primary care facilities. As a result of the unavailability and inaccessibility of these rehabilitation services, many stroke survivors were referred to HCBC. However, these services were delayed, fragmented and brief.^44^ Less than 50% of the assistive product needs were met.^44^

In the Breede Valley subdistrict (Figure 1; population 176 578) where this study was conducted, acute care was offered through the district hospital, with ambulatory rehabilitation services provided by a roving team of three therapists at the primary care facilities. Home- and community-based care was provided in 10 municipal wards through a non-governmental organisation by CHWs who lived in the wards that they served. Undergraduate physio- and speech therapy students from Stellenbosch University Rural Clinical School were placed in two of the wards and often accompanied the CHWs.

The wards were lower socio-economic communities. Residents were dependent on public healthcare services and lived in low-cost housing developments or informal settlements in urban, peri-urban and rural settings.

### Study population and selection of participants

The study population included all stroke survivors receiving HCBC, their caregivers and CHWs in November 2014.

Four focus groups of 10–16 people each were planned for stroke survivors and caregivers, giving a total sample of 40–64 people. The sampling purposefully selected stroke survivors and caregivers who were typical of those receiving HCBC services in the district. Twenty-seven pairs of stroke survivors and caregivers were purposively selected by the care coordinators and researcher, using the following criteria: participants must be from all the municipal wards, at least 6 months post-discharge, a mix of genders and ages, reliant on a caregiver and able to share their experiences in a group setting. Invitations were extended and participation confirmed via the care coordinators. Data saturation was determined by the emergence of new themes in the final FGI. If no new themes were identified, then no further focus groups would be planned.

All 44 CHWs who had experience of delivering HCBC to stroke survivors were invited by the care coordinators to participate in four FGIs. No sampling was planned as all eligible CHWs were included in the invitation.

### Data collection

Four focus groups for stroke survivors and caregivers together and four focus groups with CHWs were conducted. The FGIs were held in community centres in each ward to facilitate access. The care coordinators and two or three CHWs supported the researcher in the caregiver and stroke survivor groups by observing, providing physical assistance, signing consent forms and translating or clarifying unclear speech. The researcher independently conducted the FGIs with the CHWs. The researcher used semi-structured interview guides to explore the perceived needs of stroke survivors, caregivers and CHWs in the immediate post-discharge period: how did they manage and know what to do, what was difficult and what did they need help with? More specific questions explored facilitators, barriers and safety concerns. With Afrikaans being the most commonly understood and spoken language, the FGIs were conducted in Afrikaans, except in two groups where some participants contributed in English and isiXhosa (with immediate translation into Afrikaans or English by the CHWs). The FGIs lasted approximately 90 minutes and were audio recorded (in addition to field notes).

### Data analysis

The thematic analysis approach provided a structured approach to identify and organise recurring patterns of meaning across data sets and to provide insight into these patterns or themes in order to explore the research question. Thematic analysis was able to describe in-depth the phenomena of interest across the data set as well as interpret underlying meanings, assumptions and ideas.^46^

Data analysis followed five stages of the framework method.^47,48^ The audio files were transcribed verbatim in Afrikaans and English by a research assistant. Transcriptions were thematically analysed using Atlas-ti® software. During familiarisation, data were checked for accuracy by listening to the recordings and reading the transcripts whilst identifying emerging ideas and themes. During the development of the thematic index, codes were inductively identified from the transcripts and organised into categories related to the study objectives. All transcripts were then coded. During charting, data from codes in each category were collated together in separate documents across all the FGIs. These charts were used to identify themes and the range of experiences and opinions within themes as well as relationships between themes.

### Trustworthiness

Rigour for four principles of trustworthiness^47^was achieved as follows:

Credibility: The presence of familiar care coordinators and CHWs assisted the researcher in establishing rapport and trust with the focus group participants. The researcher engaged in-depth with the focus group participants through eight FGIs. To validate the final interpretation, the transcripts were checked by the researcher, care coordinators and CHWs who attended the FGIs. It was not possible to do this with caregivers and stroke survivors. To strengthen the credibility of the analysis, the thematic index and interpretation were reviewed by the researcher’s supervisor. Data were triangulated^49^ between two groups of respondents.

Transferability: Detailed description of the participants, study setting, and findings will allow others to decide on the transferability of findings to similar settings.

Dependability: Dependability was supported by the detailed description of the methods and the ability to audit the process of data collection and analysis with the help of Atlas-ti.

Confirmability: The researcher’s credentials and relationship with the participants were described and the researcher remained aware of his or her own subjectivity during data interpretation. The researcher, care coordinators and CHWs, who assisted in the FGIs, kept field notes and journals.

## Findings

Of the 54 caregivers and stroke survivors invited, 1 stroke survivor and 6 caregivers could not attend because of illness, transport and personal reasons, whilst 3 of the 44 CHWs invited were unable to attend as a result of training commitments. Participant profiles are detailed in Tables 1 and 2. To protect participants’ anonymity and to maintain confidentiality, age and gender were not used as identifiers with quotes from participants.

**TABLE 1:** Profile of stroke survivors and family caregivers in focus group discussions per focus group.

Characteristics	Focus group 1	Focus group 2	Focus group 3	Focus group 4
**Stroke survivors**				
**Gender**				
Male	0	6	4	2
Female	5	2	4	3
**Age range (years)**				
40–49	0	3	0	1
50–59	3	2	3	1
60–69	2	3	4	2
70–79	0	0	1	1
**Time since stroke**				
< 1 year	2	3	5	3
1–3 years	1	1	1	1
3 years+	2	4	2	1
**Total**	**5**	**8**	**8**	**5**
**Family caregivers**				
**Gender**				
Male	0	0	0	1
Female	4	5	6	5
**Age range (years)**				
20–29	0	0	1	2
30–39	1	1	2	1
40–49	2	3	2	2
50–59	1	1	0	1
60–69	0	0	1	0
**Relation to stroke survivor**				
Partner or spouse	0	3	2	2
Son or daughter or in-law	2	1	2	3
Other family member	1	1	2	1
Friend	1	0	0	0

**Total**	**4**	**5**	**6**	**6**

**TABLE 2:** Profile of community health workers in focus group discussions per focus group.

Characteristics	Focus group 5	Focus group 6	Focus group 7	Focus group 8
**Gender**				
Male	0	1	0	0
Female	17	10	7	6
**Age range (years)**				
20–29	6	3	2	1
30–39	6	5	3	2
40–49	5	2	1	3
50–59	0	1	1	0
**Years working**				
< 1 year	1	1	1	1
1–3 years	11	5	3	3
3 + years	5	5	3	2

**Total**	**17**	**11**	**7**	**6**

The findings are presented and discussed under four main themes, namely, need for emotional support, figuring it out by yourself, impact of contextual factors and implications of organisation of services. Eleven sub-themes were identified and are presented within these four main themes. At the core was the stroke survivor’s needs, which impacted both the caregivers’ and CHWs’ needs. Similarly, caregivers’ needs impacted CHWs’ needs.

### Figuring it out by yourself

Not having had any rehabilitation or training since the stroke, all groups had little knowledge on stroke and how to care for the stroke survivor. All participants felt they were left to figure out things for themselves.

#### Stroke information and education

The overall knowledge, uncertainty and simplistic understanding of stroke, its risk factors, causes, symptoms, complications, recovery and treatment were poor:

‘The stroke came with the high blood [*pressure*] and sugar [*diabetes*]. This is what we know now. We should not eat salt or sugar.’ (Caregiver, FG-4)

Caregivers felt inadequately equipped to manage complications such as pain, stiffness (spasticity), blood glucose levels and seizures. They valued training and written information. The CHWs perceived caregivers and stroke survivors to often be unsure of how to use their medication. The CHWs also had questions on stroke and its consequences:

‘I want to learn more. How to handle that patient. I see my patient is very stiff. How do I lift him and how do I move him?’ (CHW, FG-6)

#### Caregiving and community health workers knowledge and skills

Caregivers lacked skills and knowledge and were overwhelmed and intimidated by fear and uncertainty. They worried about hurting or injuring the patient or themselves. They felt abandoned, with no one to turn to. The overwhelming chorus from caregivers was:

‘I did not know. There was no one to ask. I had to figure it out by myself.’

A wife whose husband came home after 3 months in hospital explained:

‘… [*H*]how must you feel? The one day he is still fine … And now, suddenly you have to look after someone who is bedbound for life! It was a completely different experience for me! I mean, to clean him on the first day! He couldn’t talk. I had to figure out what he wanted to say … I did not know how to help him, I had to figure it out all by myself … Caregiver, FG-3)

Although caregivers were eager to receive training from CHWs, CHWs themselves felt ill-equipped and equally overwhelmed and alone:

‘If I get there, what am I actually supposed to do?’ (CHW, FG-6)

On your own! How can I make things easier? You need to figure out ways on your own – what works for you and what works for the him [*stroke survivor*].’ (CHW, FG-5)

In contrast, the positive impact of early training and ongoing support was emphasised by one of the few caregivers who had received training. She was positive about her ability to cope and comfortable taking on caregiving:

‘So they taught me how to work with him. Then he came home. At home, they still came – the ‘physios.’ They still supported me with him. They were very good. They helped me really well. In the end – I didn’t have to take this – get used to it by myself – but the ‘physios’ helped me … until I was used to it.’ (Caregiver, FG-4)

Apart from basic caregiving skills, problems with continence, communication, eating, drinking, cognition and behaviour heavily increased physical and emotional caregiving burdens:

‘She can’t talk. We don’t know what she wants. All the time we try to think what she wants, because she cannot tell us. It is very painful.’ (Caregiver, FG-2)

‘She washes herself, but she doesn’t do it right … She just continues [*squeezing the cloth*]. She tries, to, but it is slow. If I do it, it goes faster.’ (Caregiver, FG-1)

Cognitive and behavioural problems were particularly poorly understood and managed by both caregivers and CHWs. One family reported that a daughter, who was the primary caregiver, was kicked out of the house by the mother who had cognitive problems. Stroke survivors were labelled as uncooperative, lazy or difficult, leading to interpersonal conflict:

‘I can’t turn her over … She’s heavy. Stiff. It feels like she doesn’t work with me. She grabs here. She grabs there.’ (Caregiver, FG-1)

‘But she doesn’t want to [*cooperate*]. She is very lazy. She does not cooperate. If I say, let’s go sit outside, it feels like I can hit her with a stick. She doesn’t want to.’ (Caregiver, FG-4)

Injury risk escalated in this group, with stroke survivors falling out of bed or when trying to rise from bed unaided. One stroke survivor suffered burn wounds after setting the bed alight. Catheters were pulled out and diapers ripped off:

‘He refuses to wear a diaper. It is really demanding on his wife. Everything comes out in the bed. And she must just clean. She cannot put on the diaper. Oh no! He is too difficult!’ (CHW, FG-7)

#### Incontinence and toilet management

Management of incontinence and toileting was identified in all FGIs as a dire need affected by multiple factors. Weakness, poor balance and dependence resulted in a heavy physical care burden:

‘In the beginning it was difficult. She struggled to sit. To go to the toilet – now that was too difficult!’ (Caregiver, FG-1)

Contextual factors such as indoor accessibility, particularly size and layout of the bathroom or toilet and lack of environmental-assistive devices such as rails further increased the care burden:

‘We [*2 people*] carry Granny to the toilet. The wheelchair doesn’t fit.’ (Caregiver, FG-2)

‘We have to hold him [*on the toilet*]. There is no place for him to hold onto. We struggle …’ (Caregiver, FG-2)

Having only outdoor toilet facilities often resulted in stroke survivors wetting or soiling the bed or themselves, particularly when there was urgency or poor sphincter control:

‘She can’t keep it in. Now we use the bucket next to the bed, because it is a long way to the toilet. Particularly when her tummy is a bit runny. Then it is sometimes very difficult to get to the toilet in time.’ (Caregiver, FG-3)

Those with outside toilets often employed unsafe strategies to avoid going to the toilet, including deliberately dehydrating themselves.

Dependence trumped privacy, adding yet another dimension of emotions such as anxiety, embarrassment, awkwardness and apprehensiveness. Stroke survivors often delayed the call for help until it becomes too late:

‘For them it’s a problem going to the toilet. It is very difficult to take the person to the toilet. Look, my mother got very frustrated if she needed to go to the toilet. Very frustrated. She did not always ask. Maybe, I was busy somewhere in the house. Then she does not want to bother me.’ (Caregiver, FG-3)

Except for the occasional small supply of linen savers, health services did not supply commonly needed incontinence devices such as diapers, mattress protectors, urinals, bedpans and commodes. Because of poverty, families resorted to low-cost alternatives such as plastic bags and newspapers for mattress protection and 20-liter paint buckets as commodes. Some stroke survivors did not have a mobility device to reach to the toilet.

Both family members and CHWs were equally uncomfortable and ill-prepared in dealing with continence matters:

‘Then he soils himself – and they did not explain to me how to handle him at home. Where to touch, how to turn, how do I get him to the toilet! … I did not know how to help him, I had to figure it out all by myself … There was no [*bed*]pan, there was no bottle. Those things and … It was very difficult for me.’ (Caregiver, FG-3)

‘She was wet and soiled, and we had to change her. O! It was difficult. I was so … I mean … This was my first. It was very fresh. It was huge … a pile! Struggled for half an hour. One still learns … One still learns. We did not know what we were doing … We need such training, because we looked really silly.’ (CHW, FG-5)

### Need for emotional support

More than 6 months following the stroke, the need for emotional support was high, as both stroke survivors and caregivers were still trying to deal with the devastating aftermath of the stroke.

#### Emotional support of stroke survivors

Stroke survivors and family caregivers had a profound sense of loss. Both groups expressed pain, sorrow, despair, frustration and anger about the loss of independence and function:

‘I’m used to do my own work. I am used to looking after myself. I cannot handle this. It is very difficult.’ (Stroke survivor, FG-1)

Some stroke survivors expressed feelings of depression and suicidal tendencies:

‘I want to step in front of a car.’ (Stroke survivor, FG-3)

Overwhelmed by their own emotions and the caregiving burden, caregivers struggled to provide emotional support to stroke survivors. They often experienced negative feelings such as impatience, frustration and anger, which further increased the stroke survivor’s sense of burdening his or her families:

‘People become difficult with you. I see it where I live. It’s my own sister, but sometimes she treats me like – it seems like I am a stranger …’ (Stroke survivor, FG-3)

The CHWs recognised the need to support caregivers and stroke survivors but felt ill-equipped, emphasising the need for training:

‘We see that the patient isn’t in a good condition today, but when you ask her: “What is the problem today? It seems you are not looking good”. She says: “No, I’m fine”. But you can see there’s something wrong.’ (CHW, FG-8)

The CHWs suggested the appointment of dedicated stroke counsellors like the HIV or AIDS and TB counsellors working at primary level. The lack of appropriate assistive products and the inability of caregivers to safely assist survivors compounded the loss of function and confined some stroke survivors to bed, leaving them feeling isolated and abandoned and thereby further increasing the need for emotionally supporting the stroke survivors.

#### Recovery from stroke

Stroke survivors yearned for recovery. A chorus of only wanting to be able to use their hands or legs, to walk and to take care of themselves echoed through all the FGIs. They expected to recover fully and thought that more exercise would lead to recovery and did not anticipate living with a disability, portraying both denial and poor knowledge of the consequences of stroke and likelihood of recovery:

‘I first want to be healed again.’ (Stroke survivor, FG-1)

‘I just want her to walk again. She must just be normal again.’ (Caregiver, FG-2)

‘To exercise my arm and leg so I can do things as before.’ (Translated by CHW for stroke survivor, FG-3)

Their continued dependence resulted in low self-worth and feelings of guilt for burdening others:

‘You feel like a throw-away doll and a burden on others.’ (Stroke survivor, FG-3)

#### Caregiver strain and emotional support

The burden on caregivers caused stress and anxiety, and most caregivers struggled to balance caregiving demands and other responsibilities:

‘I now look after the auntie. But I need to go home and also cook there as well. I must make sure that I am home before the school day ends and the kids come home.’ (Caregiver, FG-1)

Many caregivers lacked support from other family members, increasing their burden and strain. Caregivers could not single-handedly provide sufficient supervision, leading to adverse incidents:

‘Now I see it like this. If they [*caregiver’s sisters*] looked after her, she wouldn’t have fallen. It means that I cannot turn myself around.’ (Caregiver, FG-1)

The CHWs recognised the need to support the caregivers. However, for them support generally centred on practical solutions, such as trying to involve family members, setting up caregiving rosters or admitting the stroke survivor for a short period of relief care. They motivated caregivers through talking to them, not being demanding, yet emphasising their role in supporting them to care for the stroke survivor:

‘We always tell them: If there are more families, have those families relieve you, because you have been busy with that person for such a long time. Now you become irritated and tired of that person. Set up times. You come in the week. I come over weekends. Then that person does not feel the burden that much.’ (CHW, FG-5)

The CHWs advocated for community support groups for both caregivers and stroke survivors.

### Impact of contextual factors

Both environmental and personal contextual factors (as defined by the International Classification of Functioning, Disability and Health^50^) impacted the function and care of stroke survivors.

#### Environmental factors

The impact of architectural barriers has been detailed earlier. Service limitations, particularly the lack of assistive products, not only impacted incontinence management but complicated all care tasks and prolonged dependence, including mobility, communication, eating, drinking and self-care. Knowledge of assistive products and simple home modifications was poor in all groups. Expectations to receive products through services were low and caregivers functioned with a pragmatic approach:

‘Every day, they tried their best to get her to the living room so that she could sit with them. So, every day, they stood together and carried her.’ (Translated for caregiver by CHW, FG-6)

Family dynamics varied greatly. Many caregivers had good support from other family members. In contrast, some families were dysfunctional with neglect, abuse and alcoholism, risking the care and safety of stroke survivors. These stroke survivors were not washed, dressed or transferred from bed and were often wet or soiled, resulting in bedsores:

‘He [*the stroke survivor*] is bed bound … He lives at home. The children just put food down and leave again. Nobody worries about him … The social worker is full. What must you do in this situation? Exactly what must you do for him, how now, what now? Where do you go? Who do you go to? Who do you report to? All those things.’ (CHW, FG-6)

With professional services and support structures absent, CHWs were desperate, with some taking on additional care duties in their spare time, such as washing and dressing the stroke survivor, cleaning their rooms and preparing meals. In some cases, CHWs felt that the stroke survivor was only tolerated for their disability or other grant. In this low socio-economic context, poverty was rampant. Besides the loss of income from stroke survivors who had worked before, some caregivers had to give up employment to take on the caregiving role, adding to the feelings of loss and the financial burden. The CHWs were confronted with the immediate physical needs of families and stroke survivors. They needed information on how to access food assistance, clothing, social grants and other basic needs for their clients.

#### Personal factors

Dependence, and particularly continence care, shattered traditional cultural roles, especially where circumstances forced males into the caregiving role for their mothers or wives. The CHWs experienced similar cultural problems where men did not want to be washed by female CHWs:

‘I’m having a problem. We have one man … It is a culture thing, because in our culture, we are not allowed to wash a man.’ (CHW, FG-8)

### Implications of organisation of services

The organisation of health and rehabilitation services failed to meet the needs of stroke survivors, caregivers and CHWs.

#### Need for therapy

All groups wanted access to therapy services and rehabilitation exercises, generally referring to all therapies as physiotherapy. Therapy and exercises were viewed as the key to improvement:

‘It helps a lot. He could not eat by himself. The “physio” gave him exercises and taught him to become left-handed. It’s still a struggle. His speech is improving. He could not eat. And the leg too. The exercises help a lot.’ (Caregiver, FG-3)

As a result of limited service capacity and long waiting times for appointments, stroke survivors had very limited access to therapy. This was further compounded by transport barriers such as inaccessibility, cost and unavailability. Without training, many stroke survivors felt compelled to learn and solve problems by themselves:

‘You rely on yourself. Help yourself …’ (Stroke survivor, FG-3)

Dependence was often equated to laziness by caregivers and CHWs alike. However, some CHWs recognised the need for training:

‘Not all of them are lazy. They did not get the training. They were used to do things themselves. Now they are experiencing another life now. No one has told them how to do things [*now*] they have the stroke. I don’t think all of them are lazy. Some, they don’t know.’ (CHW, FG-8)

There was a strong desire for home-based therapy services like those delivered by therapy students in two wards. Although mostly positively received, their services were also experienced as unreliable, disruptive and fragmented, often ending without explanation. Student roles and functions were not always clear, resulting in unmet expectations when placements did not include therapy. There was a plea for continuity of care by CHWs and caregivers:

‘Now, just give us our own little team. I’ve had them before at home. If we can only get them. … This week it is this team. Next week you see new faces. If you think you just get used to one and you can share secrets … then you look into a new face! “Hello! I’m so and so. And all those things”. No! We know they [*CHWs*] are here in [*ward*]. They know our backgrounds. Teach them about our needs.’ (Caregiver, FG-4)

With home-based therapy not a realistic expectation in this context, caregivers’ receptiveness to assist with home exercise programmes was met with mixed responses. Most were positive about the benefits of home exercises but concerned about finding time, whilst others felt overwhelmed by yet another responsibility:

‘I just have to try to make time for it [*exercises*] … But the time I have is a bit limited. But the better he gets, the easier things are for me … (Caregiver, FG-3)

‘I don’t have time. I’m honest. I have five children. Once I am done helping her – washed and cleaned – there is no help … I have three sisters around. But no-one helps me. They won’t even empty the bucket. It tires me.’ (Caregiver, FG-1)

Caregivers thought CHWs should do the exercises in order to avoid conflict between the caregiver and stroke survivor, especially when survivors were uncooperative. This resonated with stroke survivors’ preference to receive exercises from the CHWs rather than their caregivers:

‘She says that they can train the family and children to give “physio” at home, but they will get tired. The person will get tired and say: “No! I keep teaching you. But you still can’t walk!” They will scold them. They will not have the same patience and enthusiasm to help them. You should rather teach us [*the CHWs*], the care workers, to work with them.’ (Translated by CHW for stroke survivor, FG-4).

The CHWs also cautioned against adding to the caregiver burden. They were positive about their role in teaching and supervising home exercises, particularly to provide continuity with student or formal therapy sessions:

‘Like now, now they are gone, they only start again in February. In the meantime, they can’t or don’t exercise. Then it seems the physios [*students*] must start again from scratch … And we could have been there!’ (CHW, FG-7)

#### Organisation of home-based services

The CHWs experienced a fragmented healthcare system, which failed to support the stroke survivor, caregiver and CHW. Recognising how delayed referrals perpetuated dependence, CHWs advocated for immediate referral to home-based care on discharge:

‘Now, if they send the referral after 6 months, they [*stroke survivors*] are at home, the patient is used to sitting in the bed and staying in bed the whole day.’ (CHW, FG-8)

Delayed referrals resulted in family members approaching CHWs directly in the community. The CHWs would start working with the family before receiving an official referral or care plan from their supervisors, ultimately putting themselves and the services at risk. Furthermore, CHWs’ rehabilitation scope of practice and their role is not well defined, resulting in conflicting expectations from both caregivers and CHWs:

‘They leave their responsibility to you. So they tell themselves that the patient is not their responsibility. It’s yours, because it is your work.’ (CHW, FG-8)

In some cases, the CHW’s visit brought the only relief to caregivers who then wanted to take a break and make demands:

‘They demand that the patient should be washed. This must be done. And they sit on their back.’ (CHW, FG-5)

#### Professional support

The community health workers reported that their role was not acknowledged or recognised by healthcare professionals and that they were not seen as part of the team and not consulted or informed of treatment planning. This not only impacted their credibility with families but also made them feel awkward when families had questions. They felt isolated, frustrated and disempowered by dysfunctional referral systems and a lack of feedback:

‘Your hands are cut off. You can write letters and send with the patients, or we have the green notebook we send along. I’ve made so many notes … Then when the patient returned, they haven’t done anything. So you really feel helpless.’ (CHW, FG-7)

The community health workers had varied support from and interaction with therapists and students. Some CHWs asked students to obtain information on their clients. They were desperate for links to and support from therapists, yet they were unsure what support they could expect:

‘I don’t know what would be available from the therapists so that I can ask them and help all of us. And then teach the families at home.’ (CHW, FG-8)

Those who received informal training found the lack of a uniform approach confusing but viewed it as something they just had to accept:

… [*B*]but everyone does it their own way … Yes! [*Laughter*] We get a bit confused.’ (CHW, FG-7)

### Ethical consideration

Ethical clearance (S13/09/158) was obtained from SU Health Research Ethics Committee (HREC) and permission obtained from the Provincial Health Research Committee (RP 072RP2014).

## Discussion

**FIGURE 2:**
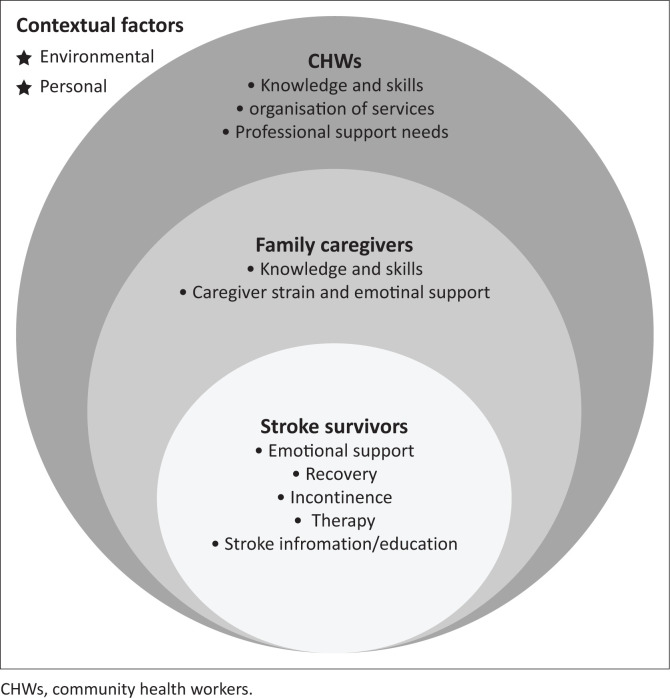
Summary and overlap of key themes emerging from three groups.

All three groups in this study felt they had to figure things out alone. There was an overwhelming need for emotional support, knowledge and skills training. Commonalities with stroke survivors and caregivers from better resourced settings, and important differences are explored below. The key findings are summarised in Figure 2 in a way that also starts to reflect on the implications for the design of a training package to address the needs of stroke survivors, caregivers and CHWs.

Whereas stroke survivors typically grapple with practical problems of integration and participation after rehabilitation, there was little cognisance of how to live with a disability in this group. Negative emotions, anxiety, depression and unrealistic expectations for recovery dominated amongst both stroke survivors and caregivers. These emotions and expectations are typically found in the immediate post-acute phase during inpatient hospital and rehabilitation care.^17,19,51,52^ This may be because the stroke survivors in this setting did not receive any rehabilitation. As long-standing anxiety and depression in stroke survivors are associated with caregiver anxiety and depression,^53,54,55^ stroke information and education, together with emotional support and counselling for stroke survivors and caregivers, are critical elements of stroke rehabilitation services.^16–18,22,53,56^ In the absence of formal rehabilitation services in this setting, targeted caregiver training should become a priority for HCBC and be supported by clinical practice guidelines.

Similar to previously reported findings, caregivers were eager to gain knowledge and skills to help them manage the care burden, particularly with respect to aspects associated with a heavier burden: dependence,^15,54,57,58,59^ incontinence,^57,59^ cognitive and behavioural problems,^53,54^ problems with eating, drinking, swallowing^54^ and communication.^54^ These needs are usually more prevalent during the acute and inpatient rehabilitation stages^14,51,56,58^ and should be the focus of the future caregiver training programme.

Contextual factors, such as poverty, architectural barriers and a lack of services and assistive products, contributed to dependence and immense indignity around self-care, toileting and incontinence management. Poor incontinence management is associated with poor quality of life^60^ and should be addressed urgently. Comprehensive incontinence management including specific bladder and bowel function assessment and treatment, medication, bowel- and bladder-training programmes, prescription of incontinence wear and products as well as toileting products can be effective in reducing caregiver strain post-discharge.^57^ Incontinence management, including identifying the need for assistive products and self-made assistive products, would be further essential elements of the training programme.

Availability of health services, ability to coordinate care and severity of stroke influence caregiver and stroke survivor expectations and needs over time.^17,56,58,61,62,63,64,65^ The needs of caregivers and stroke survivors in this study remained focused on basic care, whereas their counterparts from resourced settings focused on social participation and integration.^56,58,62,63^ In addition to limited and fragmented services, lack of support and training and poor provision of assistive products, participants in this study demonstrated lack of rehabilitation service knowledge and had low service delivery expectations. They appeared trapped in survival mode, with life centred on the care burden, rather than what is possible despite the disability. Clinical practice pathways and evidence-based practice guidelines should be developed to facilitate care coordination and promote best clinical practice.^66,67^

The need for caregiver training is at its highest prior to discharge and has been found to decrease the physical and emotional care burden, even in low-resourced settings.^10,17,18,22,56,58^ Caregivers in this study had little or no training. Assuming a caregiver role with its associated heavy physical burden requires specific intervention and support.^22,56,68,69^ Tools can assist in identifying the caregivers’ support needs and timing of interventions.^12,17,56^ A caregiver’s capacity and competency can be formally assessed and are associated with experience, knowledge, skills, physical ability, health, mental health, financial resources, informal support networks and home accessibility.^15,18,70^ Although it may not be possible to assess these factors prior to discharge in low-resource settings, an early assessment after discharge could identify at-risk families who might require more support, close monitoring or intervention by formal services. This has implications for referral systems and for the operational model of the district health and therapy teams. Monitoring and supporting caregivers would also be an essential element of the training programme.

Instead of being a safety net and a source of support, knowledge and skills, the HCBC services were delayed and CHWs lacked the necessary knowledge and skills. Contrary to the recommendations to include CHWs as part of multidisciplinary teams,^71^ CHWs functioned in isolation and were not valued or recognised as team members or key community resources. These experiences are common in HCBC programmes in SA and Africa.^72,73,74,75^ Despite these constraints, they were accepted and trusted by the community they served. An appropriate training programme will equip CHWs to train and support caregivers. The development of clinical care pathways would contribute to the coordination of care and formalise the roles of CHWs and rehabilitation professionals.

### Limitations

Practical and logistical concerns such as transport and lack of secondary caregivers influenced the composition of FGIs and resulted in caregivers and stroke survivors being in the same focus groups. This could have limited the degree of disclosure because of the presence of caregivers, stroke survivors and CHWs in the same groups. The mix of languages and cultures in two FGIs may also have had an inhibiting effect on participants. Transferability of the results will be limited to similar contexts within SA and other less resourced settings.

### Recommendations

Evidence-based clinical practice guidelines and pathways should be designed and implemented to facilitate care coordination, promote good practice and define the role and scope of both CHWs and HCBC in stroke rehabilitation, including links with formal services. The focus of a training programme for caregivers of stroke survivors in this setting should be on practical caregiving tasks, incontinence management, providing psychosocial support and identifying at risk families as well as identifying the need for and providing assistive products. With appropriate training, CHWs are an important resource at primary care level to train and support stroke survivors and caregivers.

## Conclusion

The experiences and needs of stroke survivors, caregivers and CHWs were dominated by practical caregiving and incontinence management problems and shaped by the fragmented health system and socio-economic context. In the absence of acute stroke centres and formal rehabilitation services, appropriate responsive home-based services are necessary. Community health workers can be pivotal in the training and supporting the family caregivers and stroke survivors but need appropriate training for themselves. Rehabilitation services should implement evidence-based clinical care guidelines in stroke rehabilitation services. Clinical care pathways should be developed to define the role and scope of both CHWs and HCBC in stroke rehabilitation, including links with formal services. The role of district therapists, especially in relation to the support of CHWs, should also be defined.
